# Urine microscopy as a biomarker of Acute Kidney Injury following cardiac surgery with cardiopulmonary bypass

**DOI:** 10.1590/2175-8239-JBN-2018-0133

**Published:** 2019-10-21

**Authors:** João Carlos Goldani, José Antônio Poloni, Fabiano Klaus, Roger Kist, Larissa Sgaria Pacheco, Elizete Keitel

**Affiliations:** 1 Santa Casa de Misericórdia de Porto Alegre, Departamento de Nefrologia, Porto Alegre, RS, Brasil.; 2 Universidade Federal de Ciências da Saúde de Porto Alegre, Programa de Pós-Graduação em Patologia, Porto Alegre, RS, Brasil.; 3 Santa Casa de Misericórdia de Porto Alegre, Laboratório de Análises Clínicas Carlos Franco Voegeli, Porto Alegre, RS, Brasil.; 4 Universidade Federal de Ciências da Saúde de Porto Alegre, Programa de Pós- Graduação em Ciências da Saúde, Porto Alegre, RS, Brasil.; 5 Escola de Saúde, Universidade do Vale dos Sinos, Novo Hamburgo, RS, Brasil.

**Keywords:** Acute Kidney Injury, Thoracic Surgery, Biomarkers, Lesão renal aguda, Cirurgia Torácica, Biomarcadores

## Abstract

**Introduction:**

Acute kidney injury (AKI) occurs in about 22% of the patients undergoing cardiac surgery and 2.3% requires renal replacement therapy (RRT). The current diagnostic criteria for AKI by increased serum creatinine levels have limitations and new biomarkers are being tested. Urine sediment may be considered a biomarker and it can help to differentiate pre-renal (functional) from renal (intrinsic) AKI.

**Aims:**

To investigate the microscopic urinalysis in the AKI diagnosis in patients undergoing cardiac surgery with cardiopulmonary bypass.

**Methods:**

One hundred and fourteen patients, mean age 62.3 years, 67.5 % male, with creatinine 0.91 mg/dL (SD 0.22) had a urine sample examined in the first 24 h after the surgery. We looked for renal tubular epithelial cells (RTEC) and granular casts (GC) and associated the results with AKI development as defined by KDIGO criteria.

**Results:**

Twenty three patients (20.17 %) developed AKI according to the serum creatinine criterion and 76 (66.67 %) by the urine output criterion. Four patients required RRT. Mortality was 3.51 %. The use of urine creatinine criterion to predict AKI showed a sensitivity of 34.78 % and specificity of 86.81 %, positive likelihood ratio of 2.64 and negative likelihood ratio of 0.75, AUC-ROC of 0.584 (95%CI: 0.445-0.723). For the urine output criterion sensitivity was 23.68 % and specificity 92.11 %, AUC-ROC was 0.573 (95%CI: 0.465-0.680).

**Conclusion:**

RTEC and GC in urine sample detected by microscopy is a highly specific biomarker for early AKI diagnosis after cardiac surgery.

## Resumo

**Introdução:**

Lesão renal aguda (LRA) ocorre em cerca de 22% dos pacientes submetidos a cirurgia cardíaca e 2,3% necessitam de terapia renal substitutiva (TRS). Os atuais critérios diagnósticos para LRA fundamentados no aumento dos níveis de creatinina sérica apresentam limitações e novos biomarcadores estão sendo testados. O sedimento urinário é um biomarcador que pode ajudar a diferenciar a LRA pré-renal (funcional) da LRA renal (intrínseca).

**Objetivos:**

Investigar a urinálise microscópica no diagnóstico de LRA em pacientes submetidos a cirurgia cardíaca com circulação extracorpórea.

**Métodos:**

Um total de 114 pacientes com idade média de 62,3 anos, 67,5% do sexo masculino e níveis médios de creatinina de 0,91 mg/dL (DP 0,22) tiveram amostras de urina examinadas nas primeiras 24 horas após a cirurgia. A identificação de células epiteliais tubulares renais (CETR) e cilindros granulares (CG) foi associada a desfechos de desenvolvimento de LRA conforme os critérios do KDIGO.

**Resultados:**

Vinte e três pacientes (20,17%) desenvolveram LRA pelo critério de creatinina sérica e 76 (66,67%) pelo critério de diurese. Quatro pacientes necessitaram de TRS. A mortalidade foi de 3,51%. O uso da creatinina urinária como critério preditivo para LRA mostrou sensibilidade de 34,78% e especificidade de 86,81%; razão de verossimilhança positiva de 2,64 e razão de verossimilhança negativa de 0,75; e ASC-COR de 0,584 (IC 95%: 0,445-0,723). Para o critério de diurese, a sensibilidade foi de 23,68% e a especificidade 92,11%; a ASC-COR foi 0,573 (IC 95%: 0,465-0,680).

**Conclusão:**

A identificação de CETR e CG em amostras de urina por microscopia representa um biomarcador altamente específico para o diagnóstico precoce de LRA após cirurgia cardíaca.

## Introduction

Acute kidney injury (AKI) is a frequent syndrome, especially in hospitalized patients. It is associated with increased morbidity and short and long-term mortality. Currently, it is defined as an abrupt decline in glomerular filtration rate (GFR) resulting from an injury that causes a functional or structural change in the kidney. It is recognized by an increase of serum creatinine concentration and urine output less than 0.5 mL/kg/h^1^. It occurs in diverse settings and may range from minimal elevations in the serum creatinine to the anuric renal failure and, consequently, to the necessity for renal replacement therapy (RRT). AKI is one of the complications of cardiac surgery. Pickering et al. showed an AKI frequency of 18.2% in patients who undergo cardiac surgery with cardiopulmonary bypass, and 2.1 % needed RRT dialysis. Moreover, AKI was associated with significant morbidity and mortality independent of all other factors^2^. Another meta-analysis in adult patients found an AKI incidence of 22.3% in total, being 13.6% stage I, 3.8% stage II, and 2.7% stage III, whereas 2.3% received renal replacement therapy RRT^3^.

However, AKI current criteria have been criticized due to their limitations, insensitivity for the early detection of kidney injury, and non-specificity. In order to overcome these drawbacks, several biomarkers have been evaluated for the early diagnosis and AKI risk stratification, as the combination of Interleukin-18 (IL-18) and Kidney Injury Molecule 1 (KIM-1)^4^.

Recently, a combination of two biomarkers, tissue inhibitor of metalloproteinase (TIMP-2) (2) and insulin-like growth factor binding protein (IGFBP7)^5^, was approved as a test to determine the risk of developing moderate to severe AKI in critically ill patients^6^. These biomarkers are cell cycle arrest markers and they were chosen among more than 300 candidates.

The urine sediment is an objective biological indicator for normal or pathogenic processes in the kidney and can be used as an AKI biomarker^7^. Urinary microscopy of patients with acute tubular necrosis (ATN) is classically described as containing renal tubular epithelial cells (RTEC), renal epithelial cells casts, granular casts (GC), or mixed casts, whereas sediment in patients with prerenal AKI contains only occasional hyaline casts^5^^,^ 8 ^,^ 9 ^,^ 10.

Perazella et al. evaluated the urine sediment for differentiating ATN from pre-renal AKI and showed that the RTEC and GC presence were predictive of ATN^11^^,^ 12 ^,^ 13. They also studied 249 patients with AKI and established a scoring system based on the GC and RTEC numbers that was associated with AKI stage at the consultation time and follow-up^5^.

Herein, we investigated the microscopy urinalysis as a diagnostic criteria for AKI in patients submitted to cardiac surgery with cardiopulmonary bypass, in the first 24 h after the surgery.

## Patients and methods

This was a prospective observational study performed at Santa Casa de Misericórdia de Porto Alegre. Considering an AKI frequency of 30%, we calculated a sample of 110 patients to provide the study with 80% power for detecting an odds ratio of 3 at a two-sided alpha of 0.05. Data were collected from July 2015 to March 2016.

Included participants were patients above 18 years old undergoing elective cardiac surgery with cardiopulmonary by-pass. Exclusion criterion was presence of chronic kidney disease defined by estimated glomerular filtration rate below 60 mL, or presence of proteinuria, or hematuria in pre-operative urinalysis.

Serum creatinine was measured by kinetic automatized method. For urine analysis, fresh urine samples were obtained in the first 24 h after the surgery and examined in less than 1 h. All samples were collected from urinary catheters. Urine (10 mL) was centrifuged at 1500 rpm for 5 min in a standard centrifuge, the supernatant (9.5 mL) was decanted, and the residue (0.5 mL) was resuspended by gentle manual agitation of test tubes. A single urine sediment drop was pipetted on a glass slide, and a coverslip was applied. There was no variation in glass slides or coverslips types used during the study. The urinary sediment was analyzed for RTEC and GC and recorded as present or absent. When present, the quantification was performed in the bright-field and phase contrast microscope at low power field (LPF) (x10) and high-power field (HPF) (x40).

Other elements, e.g. epithelial cells, erythrocytes, leukocytes, other types of urinary casts, and crystals were also recorded. The urinalysis readings were completely blinded to the AKI diagnosis. The equipment used for analysis was Clinitek Advantus of Siemens. The reactants straps were from Multistix 10SG, Siemens (Siemens Healthneers, Germany).

A scoring system was used consisting of adding points assigned to the number of RTECs and/or GC present in the sediment. Zero GC/LPF or zero RTECs/HPF = 0 points; one to 5 GC/LPF or one to 5 RTECs/HPF = 1 point; more than 6 GC/LPF or more than 6 RTECs/HPF = 2 points. Final score ranged from 0 to 4^5^.

Patient’s charts were reviewed for creatinine and urine output, days in the intensive care unit, and hospital length. Perioperative variables (age, sex, weight, height and comorbidities) and surgery length, clamp-cross time, and perfusion time were also recorded.

The present study was approved by the local Human Research Committee.

The categorical data are presented as percentage and continuous variables as mean or median as appropriated. Sensitivity, specificity, positive predictive value, negative predictive value, positive likelihood ratio, negative likelihood ratio, diagnostic odds ratio, Youden’s Index, accuracy, and AUC-ROC were calculated to assess the diagnostic properties of the urinary sediment as an AKI biomarker^14^. The SPSS version 22 and the MedCal Diagnostic test evaluation calculator (free version online) were used for statistical analysis.

## Results

One hundred and fourteen patients undergoing cardiac surgery with cardiopulmonary bypass were evaluated. The mean age was 62.3 years (SD 11.2), 67.5% were male. Preoperative mean serum creatinine (SCr) was 0.91 mg/dL (SD 0.22). Table 1 shows patients and surgery characteristics.

**Table 1:** Patients and surgical characteristics

Age -years, mean (SD)	62.3 (11.2)
Men	67.5 %
Body mass index (kg/m^2^)	27.99 (4.82)
Hypertension	81.58 %
Diabetes	30.70 %
PCOD	7.89 %
Peripheric vascular disease	8.77 %
Previous myocardial infarction	20.07 %
Other comorbidity	6.14 %
Type of surgery	
Coronary artery by-pass valve	67.55 %
Coronary artery by-pass and valve Aortic	13.16 %
Aortic and valve	9.65 %
Aortic and CAB	4.39 %
Atrial myxoma	1.75 %
Pre-op serum creatinine (mg/dL) mean (SD)	0.91 (0.22)
Perfusion time (min) mean (SD)	88.29 (36.06)
Cross-clamp time (min) mean (SD)	68.22 (23.67)
Surgery Duration (min) mean (SD)	314.31 (76.52)

SD: standard deviation CAB: coronary artery bypass

PCOD: Pulmonary chronic obstructive disease

According to KDIGO SCr criterion, 23 patients (20.17%) had AKI being 16 stage I, 3 stage II, and 4 stage III. According to urinary output (UO) criterion, 76 patients (66.67%) developed AKI, 20 of them also fulfilled the SCr criterion. Taking into account SCr and/or UO, 79 (69.3%) had AKI. Four patients (3.51%) needed RRT, three of them died, and one recovered renal function. One patient classified as stage II died. Mortality rate was 3.51% among all patients with AKI.

The urine sediment score zero, 1, 2, and 4 were present in 94, 13, 6, and 1 patients, respectively. The sediment score in each stage of AKI are presented in Table 2.

**Table 2:** Urine microscopy score in each AKI stage

Urine Microscopy	No AKI N (%)	AKI Stage			Total N (%)
		Stage I N (%)	Stage II N (%)	Stage III N (%)	
Score 0	78 (85.7)	12 (75)	3 (100)	1 (25)	94 (82.5)
Score 1	9 (9.9)	2 (12.5)	0	2 (50)	13 (11.4)
Score 2	4 (4.4)	1 (6.3)	0	1 (25)	6 (5.3)
Score 4	0 (0)	1 (6.3)	0	0 (0)	1 (0.9)
Total (%)	91 (79.8)	16 (14)	3 (2.6)	4 (3.5)	114 (100)

As the number of patients with scores 2 and 4 was small, we had to analyze the urinary sediment performance to predict AKI utilizing any score greater than one, shown in Table 3. The calculations based on SCr criterion or UO criterion and both were made separately.

**Table 3:** Urine Sediment Score 1 or more and development of AKI

	AKI-creatinine criterion (95%CI)	AKI-urine output criterion (95%CI)	AKI-creatinine and/ or urine output criteria (95%CI)
Sensitivity	34.78 % (16.38 - 57.27)	23.68 % (14.68 - 34.82)	22.78% (14.10 - 33.60)
Specificity	86.81 % (78.10 - 93.00)	92.11 % (78.62 - 98.34)	94.29% (80.84 - 99.30)
Positive Predictive Value	40.00 % (19.12 - 63.95)	85.71 % (63.66 - 96.95)	90.00% (68.82 - 97.35)
Negative Predictive Value	84.04 % (75.05 - 90.78)	37.63 % (27.79 - 48.28)	35.11% (31.88 - 38.47)
Positive Likelihood Ratio	2.64 (1.22 - 5.69)	3.00 (0.94 - 9.56)	3.99 (0.98 - 16.26)
Negative Likelihood Ratio	0.75 (0.55 - 1.02)	0.83 (0.71 - 0.97)	0.82 (0.71 - 0.95)
Accuracy	74.56% (65.55 - 82.25)	45.61% (36.26 - 55.21)	44.74% (35.74 - 54.34)
Diagnostic odds ratio	2.62 ( 0.90 - 7.61)	3.36 (0.92 - 12.29)	4.86 (1.06 - 22.28)
Youden’s Index	0.2159	0.1579	0.1707
AUC-ROC	0.584 (0.445 - 0.723)	0.573 (0.465 - 0.680)	0.583 (0.477 - 0.693)

**Figure 1:**
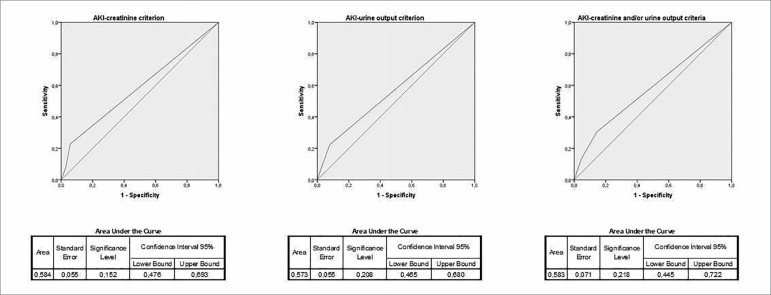
Area under the receiver operating characteristic curve of urine sediment score to distinguish AKI.

The area under the ROC-curve is showed in Figure 1. The mean peak serum creatinine concentration for AKI patients was 1.71 mg/dL (SD 0.57). Peak SCr in non-AKI was 0.92 mg/dL (SD 0.23) and AKI stage I, II, and III were 1.52 mg/dL (SD 0.37), 1.41 mg/dL (0.24), and 2.68 mg/dL (0.33). The mean time to obtain the peak serum creatinine was 27.5 h (SD 18.31) with median of 19 h. SCr in AKI stage I patients returned to baseline after 24 h.

## Discussion

The AKI incidence and need for RRT were similar to those reported in the literature^2^^,^ 3 ^,^ 15. The difference in the incidence, when considering SCr or UO criteria, was similar to that described by McIlroy et al.^16^ This difference is a problem of the current definition by KDIGO, especially related to oliguria^17^.

The urine sediment in our study showed a low sensitivity and high specificity. Schinstock et al. considered any cast as a positive and found a sensitivity of 29.6% (95%CI: 15.9 - 48.5) and specificity of 89.9% (95%CI: 86.2 - 92.7). They concluded that the presence of even one RTEC or GC per high power field has more than 90% specificity for AKI diagnosis, but it is not sensitive^18^.

A systematic review found 5 studies on the urine microscopy role in AKI differential diagnosis and outcome prediction in hospitalized patients. All studies confirmed that urine microscopy is a valuable tool for AKI differential diagnosis^9^. On the other hand, data from 7 papers on AKI related to sepsis with 174 patients did not reach a conclusion^10^.

Hall et al. studied 249 patients with AKI comparing traditional and novel biomarkers, and concluded that urine protein biomarkers and microscopy significantly improve clinical determination of prognosis. The urine sediment had an AUC-ROC of 0.66 (95%CI: 0.57-0.75) similar to NGAL, KIM-1, and IL-18^19^. Another meta- analysis including 28 studies of AKI biomarkers in cardiac surgery concluded that those markers have modest discrimination and the AUC-ROC composite values were between 0.63 and 0.72^20^.

Chawla et al. developed a cast score index and assessed its precision. The inter-observer agreement was 99.8% (SD 0.29) with the coefficient of variation of 1.24%. The index considered GC and epithelial cell casts by low power field percentage with at least one cast^21^. Whether the former criteria or GC number per low power field and renal epithelial cells per high power field, as proposed, should be used remains to be defined^22^.

The prospective design, cohort homogeneity, and AKI defined by the KDIGO criteria are strong advantages of this study. Furthermore, the urine examiners were blinded to the diagnosis. A small number of cases, single-center character, as well the inexistence of comparison with other urinary indexes or biomarkers are this study’s limitations.

## Conclusion

Urine microscopy is easily available, noninvasive, inexpensive, and needs simple equipment. In our study, the presence of renal epithelial tubular cells and granular casts had a high specificity for early AKI diagnosis. Urinary microscopy could be used in conjunction with other earlier AKI biomarkers to increase the method’s discriminatory power.
